# Coix seed improves growth performance and productivity in post-weaning pigs by reducing gut pH and modulating gut microbiota

**DOI:** 10.1186/s13568-019-0828-z

**Published:** 2019-07-23

**Authors:** Zhaolong Li, Zhongning Lin, Zheng Lu, Zhihua Feng, Qi Chen, Sufang Deng, Zhenwu Li, Youquan Yan, Zhaoyang Ying

**Affiliations:** 1 Fujian Key Laboratory of Innate Immune Biology, Biomedical Research Center of South China, Fujian Normal University Qishan Campus, Fuzhou, 350117 Fujian China; 2 Institute of Animal Husbandry and Veterinary Medicine, Fujian Academy of Agricultural Sciences, Pudang, Jin-an District, Fuzhou, 350013 Fujian China; 3 Agricultural Ecology Institute, Fujian Academy of Agricultural Sciences, Pudang, Jin-an District, Fuzhou, 350013 Fujian China; 4 Fujian Engineering and Technology Research Center for Hilly Prataculture, Pudang, Jin-an District, Fuzhou, 350013 Fujian China

**Keywords:** Coix seed, Piglets, Gut microbiota, Villi, pH

## Abstract

Coix seed has traditionally been used in traditional Chinese medicine to fortify the spleen and inhibit dampness, and has shown anticancer effects in humans. However, it is not known whether coix seed improves post-weaning growth performance and productivity, and the mechanism of interaction between coix seed and gut microbiota remains unknown. In this study, we established four groups: (i) control, (ii) antibiotic-fed, (iii) coix seed powder-fed, and (iv) coix seed extract-fed. The feeding experiment was conducted for 4 weeks. Coix seed extract significantly increased average weight gain and reduced the feed/meat ratio in weaned pigs, in addition to reducing the pH of their gastric juice. Further assays demonstrated that coix seed promotes an increase in the density and length of the gastrointestinal villi. Next, 16s sequencing of gut microbiota showed that coix seed significantly increased the abundance of phylum *Bacteroidetes* and genus *Lactobacillus* (p < 0.05) and reduced the abundance of phylum *Prevotella* (p < 0.05) in the gut microbiota. In contrast, the abundance of phylum *Bacteroidetes* and genus *Lactobacillus* decreased in the control group and antibiotic group, whereas the abundance of phylum *Prevotella* increased. Our findings indicate that coix seed improves growth performance and productivity in post-weaning pigs by reducing the pH value of gastric juice, increasing the density and length of gastrointestinal villi, and modulating gut microbiota. Thus, coix seed has good potential for use as a feed supplement in swine production.

## Introduction

Weaning transition is a critical period in the growth of piglets (Wolter et al. 2002). During this period, the digestive and immune systems are in their developmental phases, and piglets experience a wide variety of stresses, such as an abrupt shift in diet from high-fat, low-carbohydrate breast milk to high-carbohydrate and low-fat feed, which can lead to poor health and growth performance (Thexton et al. 1998). Thus, antibiotics are widely used to treat bacterial infections and stress-induced gastrointestinal dysfunction. Misuse and overuse of antibiotics increase antimicrobial resistance, which is a serious threat for human, animal, and environmental health (Stein and Dong 2006; Coffin et al. 2013; Gellin et al. 1989; Lee et al. 2000). This threat has been increasingly recognized, and several solutions have been proposed. These include reducing or limiting the use of antibiotics and strictly checking the excess of antibiotics used in animal production (Centner 2016; Spiro et al. 2011; Pettigrew 2012; Van Der Fels-Klerx et al. 2011); however, the threat still persists (Hamscher et al. 2003; Sapkota et al. 2006; Murphy et al. 2007; Ferguson et al. 2016). Herbal alternatives such as traditional Chinese medicine (TCM) are widely accepted as high-efficiency and low-toxicity “medicinal diets” that are capable of preventing certain side effects (Williamson et al. 2013; Hsiao and Liu 2010; Normile 2013; Fu et al. 2015). Moreover, the majority of TCM treatments also contain certain food components that provide benefits beyond basic nutrition (Bao 2017); therefore, some TCMs can be used in the animal industry as alternative supplements to modulate gut macrobiota and promote growth, as well as to reduce the threat posed by antibiotics to animal and human health (Gao et al. 2010; Zhang et al. 2008; Hui et al. 2011; Jin et al. 2008; Li et al. 2008; Chen et al. 2009). In addition, most TCMs are rich in polysaccharides, polyphenols, phytosterols, and lactams, which are increasingly recognized for their anti-cancer, anti-oxidant, and anti-inflammatory effects; endocrine regulation; blood pressure and fat reduction; and immune regulation (He and Dai 2011; Zhao et al. 2014; Zhou et al. 2017).

Coix seed belongs to the family Poaceae, and is widely distributed in China, Japan, Thailand, and Burma. Coix seed contains a high content of essential amino acids, lipids, proteins, polysaccharides, polyphenols, phytosterols, and lactams, and has favorable physiological and pharmacological effects, including those mentioned above. As such, it has long been used in TCM (Liu et al. 2010; Kaneda et al. 1992; Tokuda et al. 1990; Qu et al. 2016; Yu et al. 2008). Coix seed has also been used as a substitute for rice or wheat in animal feed because of its high starch, oil, and protein content (Yu et al. 2008; Weng 2013; Jin et al. 2011). However, the use of coix seed for anti-stress effects during the weaning transition process has not been reported. Only a handful of studies have shown that the beneficial effects of coix seed might be associated with gastrointestinal microorganisms (Liu et al. 2019).

In this study, we investigated the effects of coix seed on the weaning transition in piglets and its subsequent effects on gastrointestinal villi, gastrointestinal pH, and the composition of gastrointestinal microbiota. Coix seed promoted higher average weight of piglets and a lower feed/meat ratio, which was associated with alteration in the structure of the gut microbiota and gut pH. The gut microbiota is known to respond to fluctuations in dietary composition; reciprocally, changes in the microbiome have profound effects on animals, including piglets.

## Methods and materials

### Preparation of coix seed

Coix seed was purchased from Donglan Medical Co., Ltd. The seed was then dried and ground into fine powder for direct mixing with feed. The extraction process of coix seeds was as follows: (1) Extract: the dry coix seed was added to the extraction tank, soaked in drinking water at six times the weight of the drug for 12 h, and cooked twice (boiling, temperature 95–100 °C). Water was added at six times the drug weight and volume for 1.5 h each time, and the extract was filtered twice. (2) Concentration: the extract was concentrated at a relative density of 1.10 at 50 °C by mixing the filtrated extract collected in step 1. (3) Drying: dextrin (1% by weight) was added to the concentrated liquid, which was stirred evenly and dried by spraying; the temperature in the drying tower was 90–95 °C. (4) Sieve mixing: the spray-dried powder was passed through an 80–160 mesh sieve, mixed evenly, and packaged to be used in the next step.

### Study animals and treatment

In total, 40 weaned piglets (Duroc × Landrace × Yorkshire; male to female ratio = 1), with an initial average body weight of 7.315 ± 0.93 kg and weaned at 21 ± 1 day of age, were purchased from Guangming Farm and Animal Husbandry Co., Ltd. (Fujian, China). They were randomly and equally divided into four groups: the (i) control group, (ii) antibiotic group, (iii) coix seed powder group (TCMP), and (iv) coix seed extract group (TCME). The piglets were ear-labeled and their weights were recorded individually, followed by feeding with the experimental diets for 4 weeks. The basal diet was formulated to meet the NRC (2012) recommendations for the nutrient requirements of piglets. The ingredients and compositions of the basal diet are shown in Table 1. The piglets were maintained under standard light (14 h light/dark) and temperature (25 ± 2 °C) conditions. The control group (n = 10) was fed the basal diet. The piglets in the antibiotic group were provided fodder containing 1/10,000 amoxicillin powder for animals (antibiotics group, n = 10). The piglets in the coix seed powder group were given fodder containing 5/100 coix seed powder (TCMP group, n = 10). The piglets in the coix seed extract group were provided with fodder containing 1/100 coix seed extract (TCME group, n = 10).

**Table 1:** Basic diet and nutrients

Corn, %	50
Wheat peel, %	4
Soybean meal, %	30
Fish meal, %	6
Premix, %	10
Total, %	100
Digestible energy DE (MJ Kg^−1^)	13.87
Crude protein CP, %	19.5
Lysine Lys, %	1.1
Calcium Ca, %	0.9
Available phosphorus AP, %	0.45

### Calculation of average body weight and feed/meat ratio

All piglets were weighed after overnight fasting on the morning of day 1 as well as on day 15 and day 28 of the feeding trial. The feed intake of each pig was recorded daily throughout the trial to calculate the feed/meat ratio (F:G).

### Determination of gut pH value and the gastrointestinal tract villi

At the end of the 28-day trial, the piglets were sacrificed in accordance with the experimental animal procedures of the Institutional Ethics Committee/Animal Care and Use Committee, and the pH values of the stomach, duodenum, small intestine, colon, ileum, cecum, and rectum contents were measured using a pH meter (PHS-5C PH, Guangzhou, China). Tissues (1 cm) from the stomach, duodenum, small intestine, colon, ileum, cecum, and rectum were collected from the piglets and fixed for 12 h in 5 mL 4% paraformaldehyde. The fixed tissue was sequentially passed through 50%, 70%, 80%, 90%, 100%, and 100% alcohol for 35 to 45 min at each concentration and clarified using 100% C_2_H_5_OH + xylene (1:1) for 30 to 40 min. The tissues were then immersed in paraffin (1:1) for 30 min. Sections of 6–12 µm thickness were cut (average 7–8 µm), placed onto slides, and stained with hematoxylin and eosin. The sections were observed and villus length was measured under a microscope.

### Sample collection and extraction of genomic DNA from gastrointestinal microorganisms

The contents of the stomach, duodenum, small intestine, colon, ileum, cecum, and rectum were collected from the piglets, and total genomic DNA from these samples was extracted using the CTAB/SDS method. The DNA concentration and purity were monitored on 1% agarose gels. The DNA was diluted to 1 ng/µL using sterile water.

### PCR and sequencing of gastrointestinal microorganisms

The 16s rRNA sequences of the gastrointestinal microorganisms were amplified using the specific primer for 16S v3-4: ACTCCTACGGGAGGCAGCA and GGACTACHVGGGTWTCTAAT (MK351252.1). The raw sequencing data were uploaded into the NCBI Sequence Read Archive database (SRA; http://www.ncbi.nlm.nih.gov/Traces/sra/) under accession number SRP156563. All PCR were carried out with 25 µL sample volumes and 12.5 µL of Phusion^®^ High-Fidelity PCR Master Mix (New England Biolabs), 0.2 μmol forward and reverse primers, and 10 ng template DNA. Thermal cycling was started with initial denaturation at 98 °C for 10 min followed by 35 cycles of denaturation at 98 °C for 10 s, annealing at 50 °C for 40 s, and elongation at 72 °C for 50 s, and a final elongation at 72 °C for 10 min.

### Library preparation and sequencing

Sequencing libraries were generated using an NEB Next^®^ Ultra™ DNA Library Prep Kit for Illumina (NEB, USA) following the manufacturer’s recommendations, and index codes were added. The library quality was assessed on a Qubit@ 2.0 Fluorometer (Thermo Scientific) and an Agilent Bioanalyzer 2100 system. Finally, the library was sequenced on an Illumina HiSeq 2500 platform and 250 bp paired-end reads were generated.

### Data analysis

Sequence analysis was performed with the UPARSE software package using the UPARSE-OTU and UPARSE-OTUref algorithms. In-house Perl scripts were used to analyze the alpha (within samples) and beta (among samples) diversity. Sequences with ≥ 97% similarity were assigned to the same OTUs. We selected representative sequences for each OTU and used an RDP classifier to annotate the taxonomic information for each representative sequence. In order to compute the alpha diversity, we rarified the OTU table and calculated three metrics: Chao1, which estimates the species abundance; observed species, which estimates the number of unique OTUs found in each sample; and Shannon index. Rarefaction curves were generated based on these three metrics.

### Community distribution

A graphical representation of the relative abundance of bacterial diversity from the phylum to species level can be visualized using a Krona chart. Cluster analysis was preceded by principal component analysis (PCA), which was applied to reduce the dimension of the original variables using the QIIME software package. QIIME can calculate both weighted and unweighted UniFrac distances, which are phylogenetic measures of beta diversity. We used unweighted UniFrac distances for principal coordinate analysis (PCoA) and the unweighted pair group method with arithmetic mean (UPGMA) clustering. PCoA helps to obtain principal coordinates and visualize them from complex, multidimensional data. The maximum variation factor is demonstrated by the first principal coordinate, the second-largest one is demonstrated by the second principal coordinate, and so on. UPGMA clustering is a hierarchical clustering method that uses average linkage and can be used to interpret the distance matrix.

### Statistical analysis

To confirm the differences in the abundances of individual taxae between groups, Metastats software (Version:1.0) was utilized. LEfSe was used for the quantitative analysis of biomarkers within different groups. This method was designed to analyze data in which the number of species is much higher than the number of samples and to provide biological class explanations to establish statistical significance, biological consistency, and effect-size estimation of the predicted biomarkers. To identify the differences in microbial communities between groups, ANOSIM and MR PP (multi-response permutation procedure) were performed based on the Curtis dissimilarity distance matrices.

## Results

### Growth performance

**Fig. 1:**
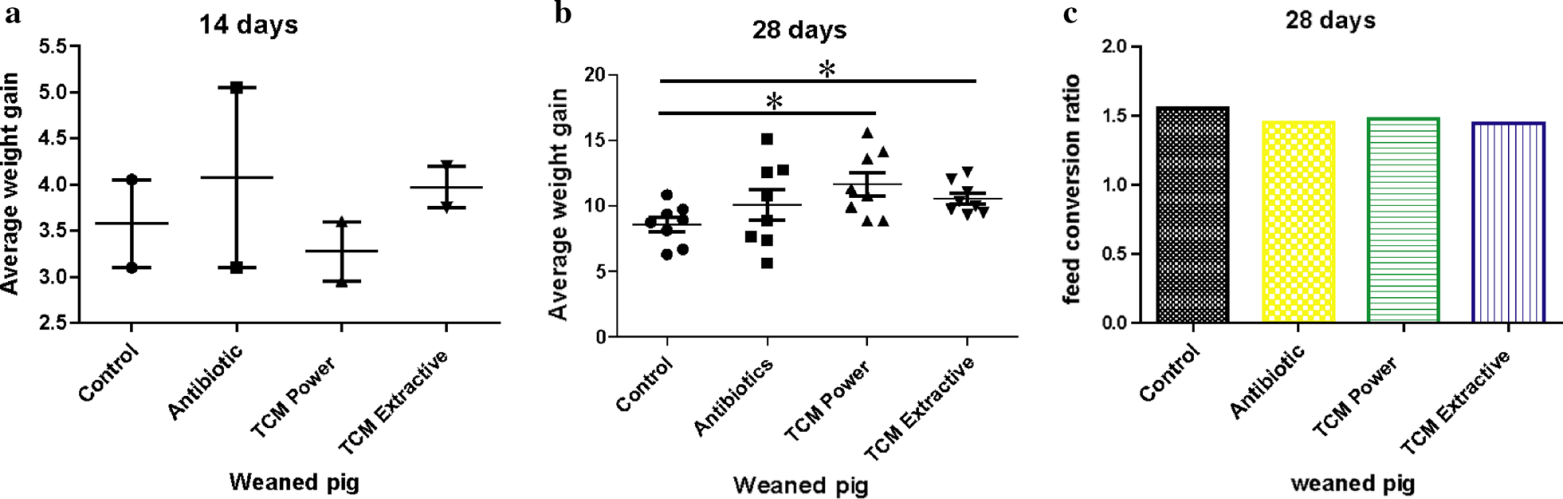
Average weight gain and feed/meat ratio of weaned pigs. **a** Average weight gain of weaned pigs at 14 days after the start of the feeding experiment. **b** Average weight gain at 28 days after the start of the feeding experiment. **c** Feed/meat ratio of weaned pigs at 28 days after the start of the feeding experiment. (*p < 0.05)

The average body weights of piglets are shown in Fig. 1. Body weight on day 28 was found to have increased under diets containing coix seed and antibiotics (*p* < 0.05). Meanwhile, the TCMP group on day 14 showed higher body weight in pigs fed diets with coix seed powder than that of pigs fed diets with either antibiotics or coix seed extracts (*p* < 0.05). However, on day 28, the highest body weight was measured in the TCME group. In addition, a lower F:G was observed in pigs fed a coix seed diet as compared to those on the antibiotic diet and control diets (*p* < 0.05).

### Gastrointestinal pH value

**Fig. 2:**
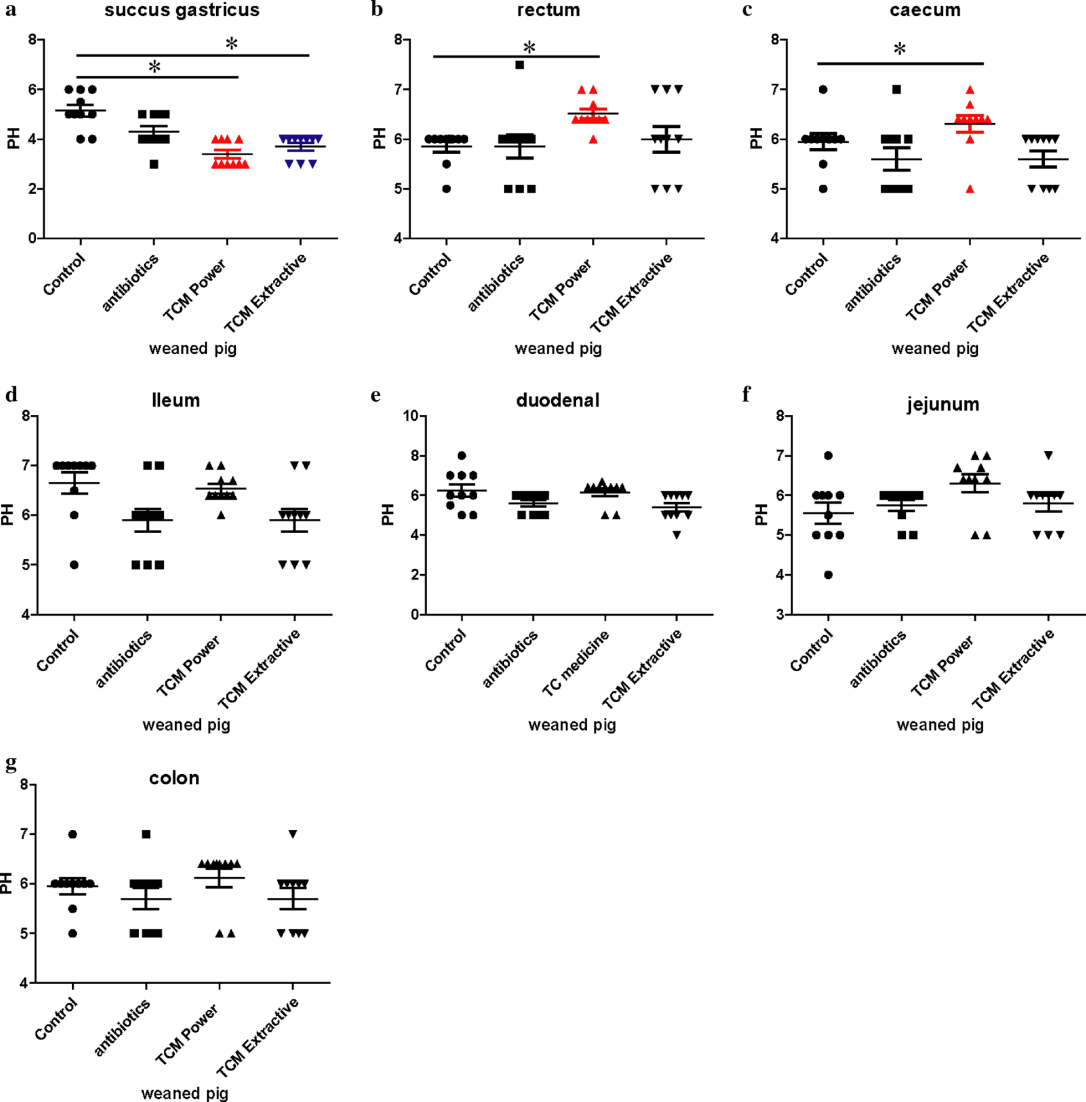
Variation in pH of the gastrointestinal content of weaned piglets on day 28. **a** pH values of the succus gastricus. **b** pH values of the rectal content. **c** pH values of the cecal content. **d** pH values of the ileal content. **e** pH values of the duodenal content. **f** pH values of the jejunal content. **g** pH values of the colonic content. (*p < 0.05)

The pH value of the gastrointestinal tract is closely related to the digestion and absorption of nutrients. It affects the microenvironment for the growth of gastrointestinal microbiota and plays an important role in regulating microbial growth. In this study, we found that the pH value of the succus gastricus in the coix seed group (TCMP and TCME) was between 2 and 3 (Fig. 2), and was lower than those of the antibiotic group and control group (*p* < 0.05). This is also the optimal activation condition for pepsin A and pepsin B. Moreover, compared with the control group and antibiotic group, the pH values of the colon, rectum, and cecum in the coix seed powder group significantly increased on day 28 (*p* < 0.05).

### Morphological changes in gastrointestinal villi in piglets

**Fig. 3:**
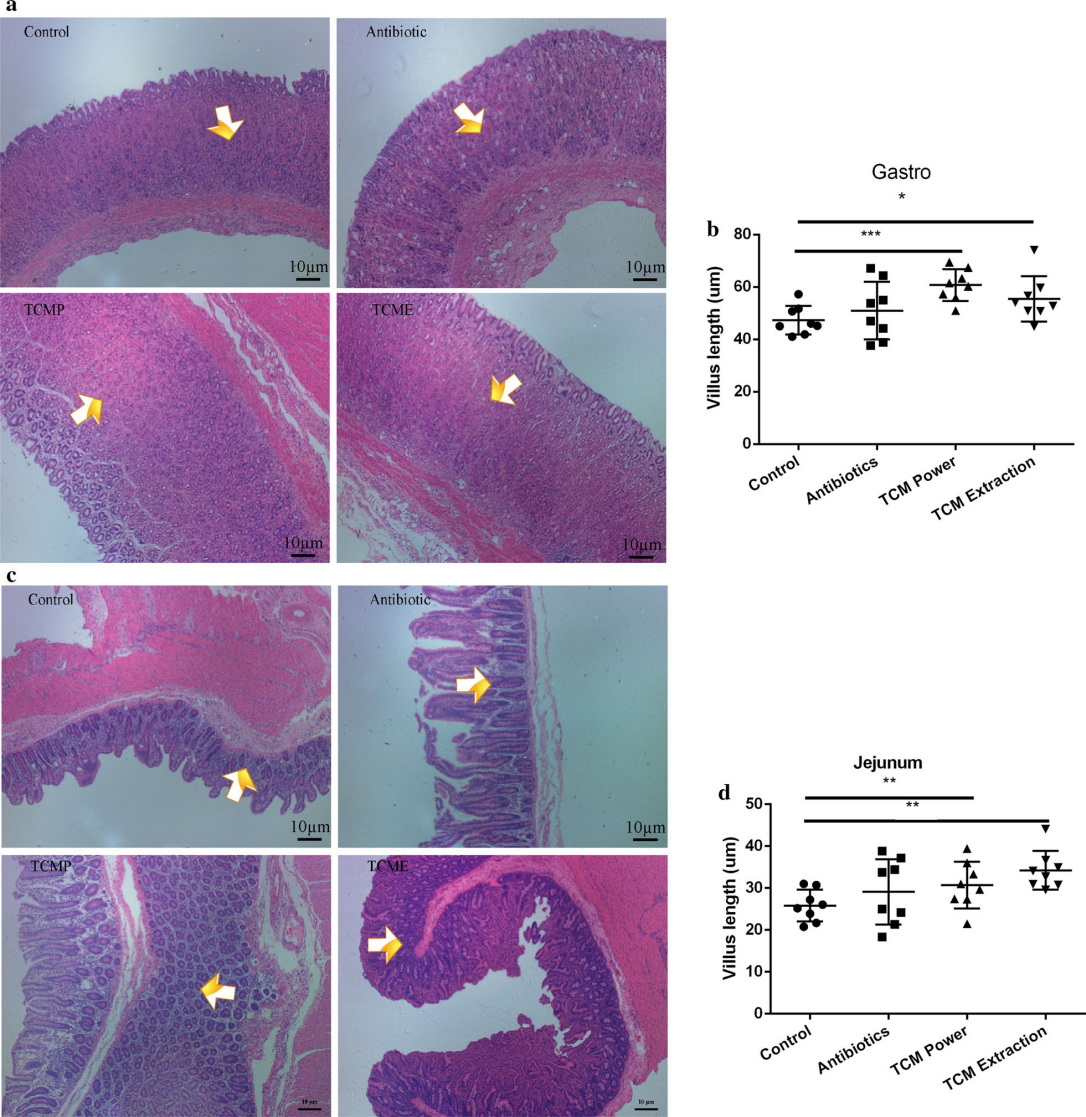
Morphological changes in the gastrointestinal villi of weaned pigs. **a** Morphological changes in gastric villi in weaned pigs. **b** Length of the gastric villi. **c** Morphological changes in the jejunum of weaned pigs. **d** Length of jejunal villi in different groups. (*p < 0.05; **p < 0.02; ***p < 0.01)

**Fig. 4:**
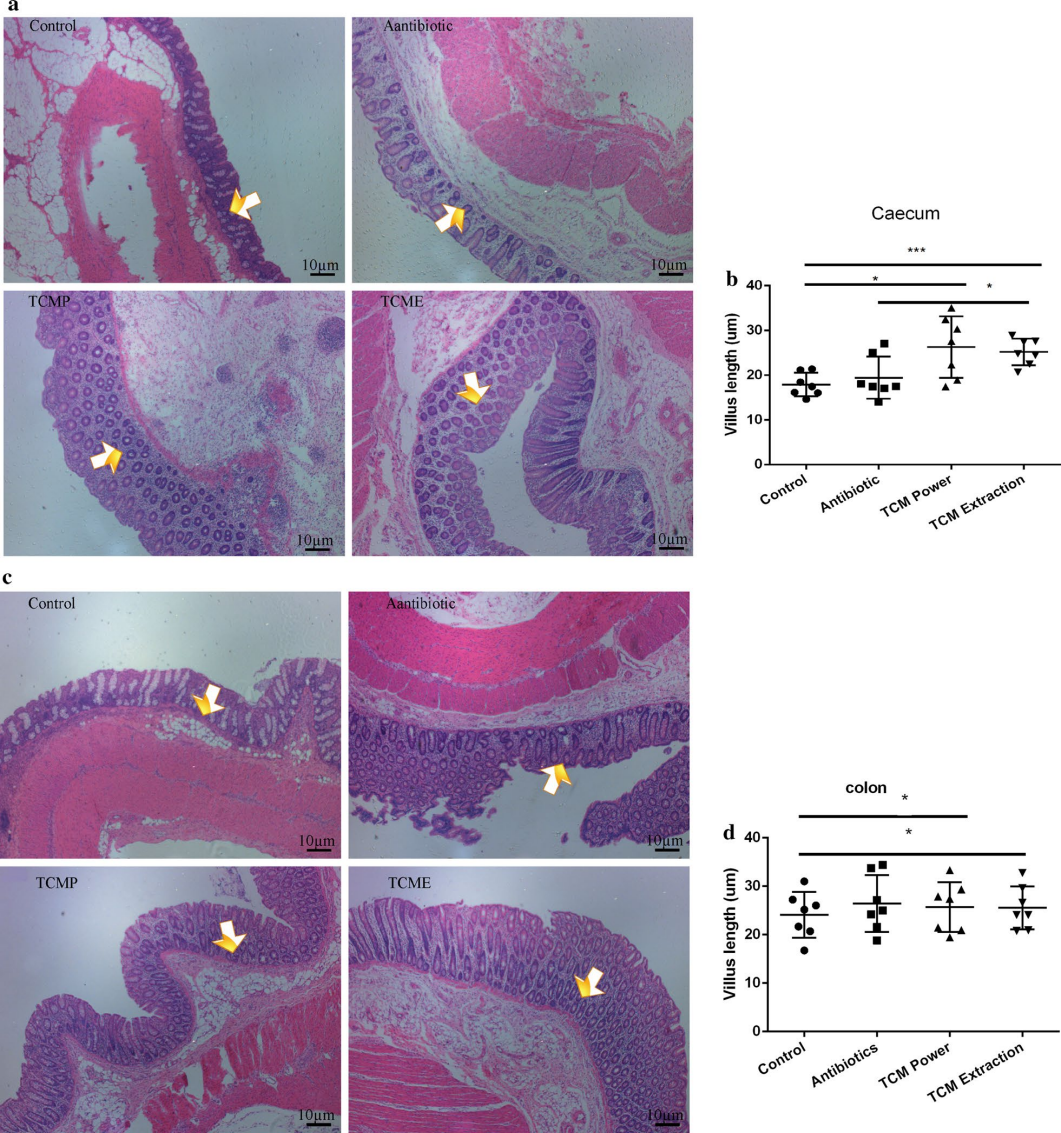
Morphological changes in the cecal and colonic villi of weaned pigs. **a** Morphological changes in the ceca of weaned pigs; **b** Length of the cecal villi. **c** Morphological changes in the colons of weaned pigs. **d** Length of colonic villi in different groups. (*p < 0.05; ***p < 0.01)

The results of the gastric villus morphology assay are shown in Fig. 3. On day 28, the villus height was more in piglets fed a coix seed diet (TCP and TCME) than in piglets fed an antibiotic diet or control diet (*p* < 0.05). Villus height in the colon, ileum, and cecum also clearly increased in piglets on a coix seed diet compared to that in piglets fed a normal diet or an antibiotic diet (Fig. 4). The maximum villus height was observed in piglets fed coix seed extract. In addition, there were some differences in villus height between the antibiotic group and control group on day 28.

### Coix seed treatment altered the gut microbiota of weaning piglets

**Fig. 5:**
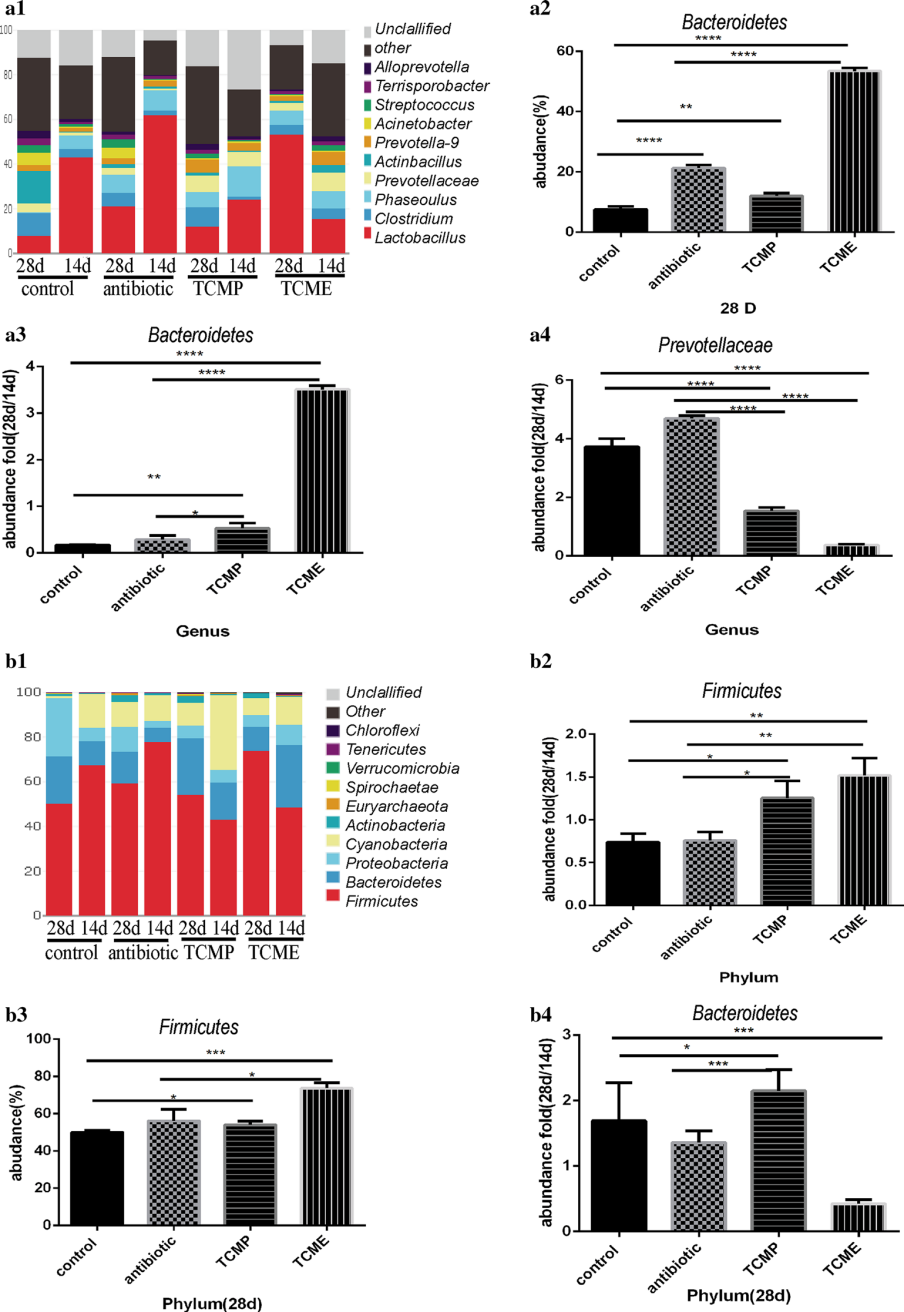
Effect of coix seed on the composition of gastrointestinal microbiota with respect to genus and phylum. **a1** Effect of coix seed on the composition of the gastrointestinal microbiota with respect to genus in weaned pigs. **a2** Abundance of genus *Bacteroidetes*. **a3** Fold abundance of genus *Bacteroidetes* (28D/14D). **a4** Fold abundance of genus *Prevotellaceae* (28D/14D). **b1** Effect of coix seed on the composition of the gastrointestinal microbiota with respect to phylum in weaned pigs. **b2** Fold abundance of phylum *Firmicutes* (28D/14D). **b3** Abundance of phylum *Firmicutes*. (b4) Fold abundance of phylum *Bacteroidetes* (28D/14D). (*p < 0.05; **p < 0.02; ***p < 0.01, ****p < 0.001)

**Fig. 6:**
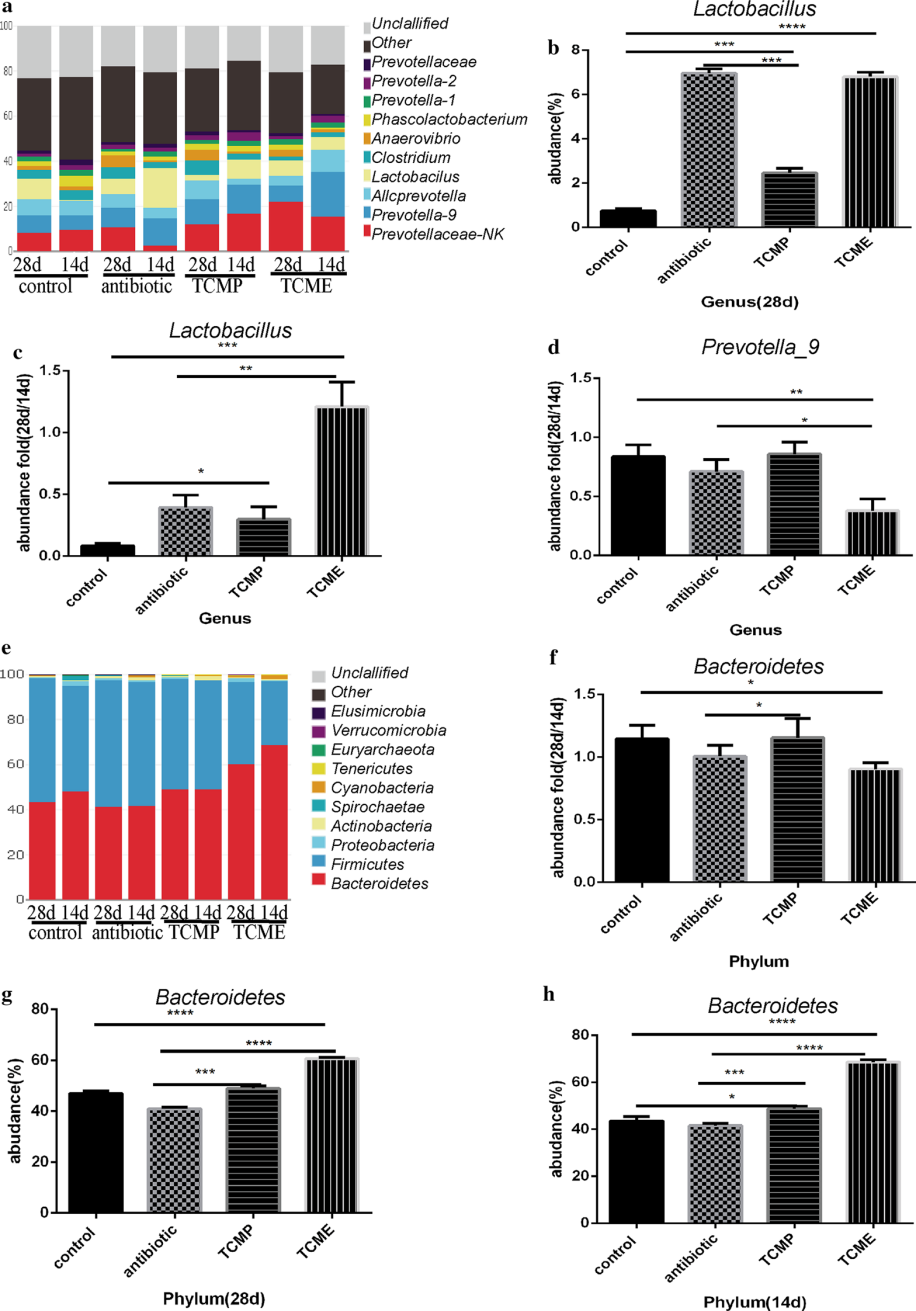
Effect of coix seed on the composition of cecal microbiota with respect to genus and phylum. **a1** Effect of coix seed on the composition of cecal microbiota with respect to genus in weaned pigs. **a2** Abundance of genus *Lactobacillus.* **a3** Fold abundance of genus *Lactobacillus* (28D/14D). **a4** Fold abundance of genus *Prevotella*-*9* (28D/14D). **b1** Effect of coix seed on the composition of cecal microbiota with respect to phylum in weaned pigs. **b2** Fold abundance of phylum *Bacteroidetes* (28D/14D). **b3** Abundance of phylum *Bacteroidetes*. **b4** Fold abundance of phylum *Bacteroidetes* (14D) (*p < 0.05; **p < 0.02; ***p < 0.01, ****p < 0.001)

**Fig. 7:**
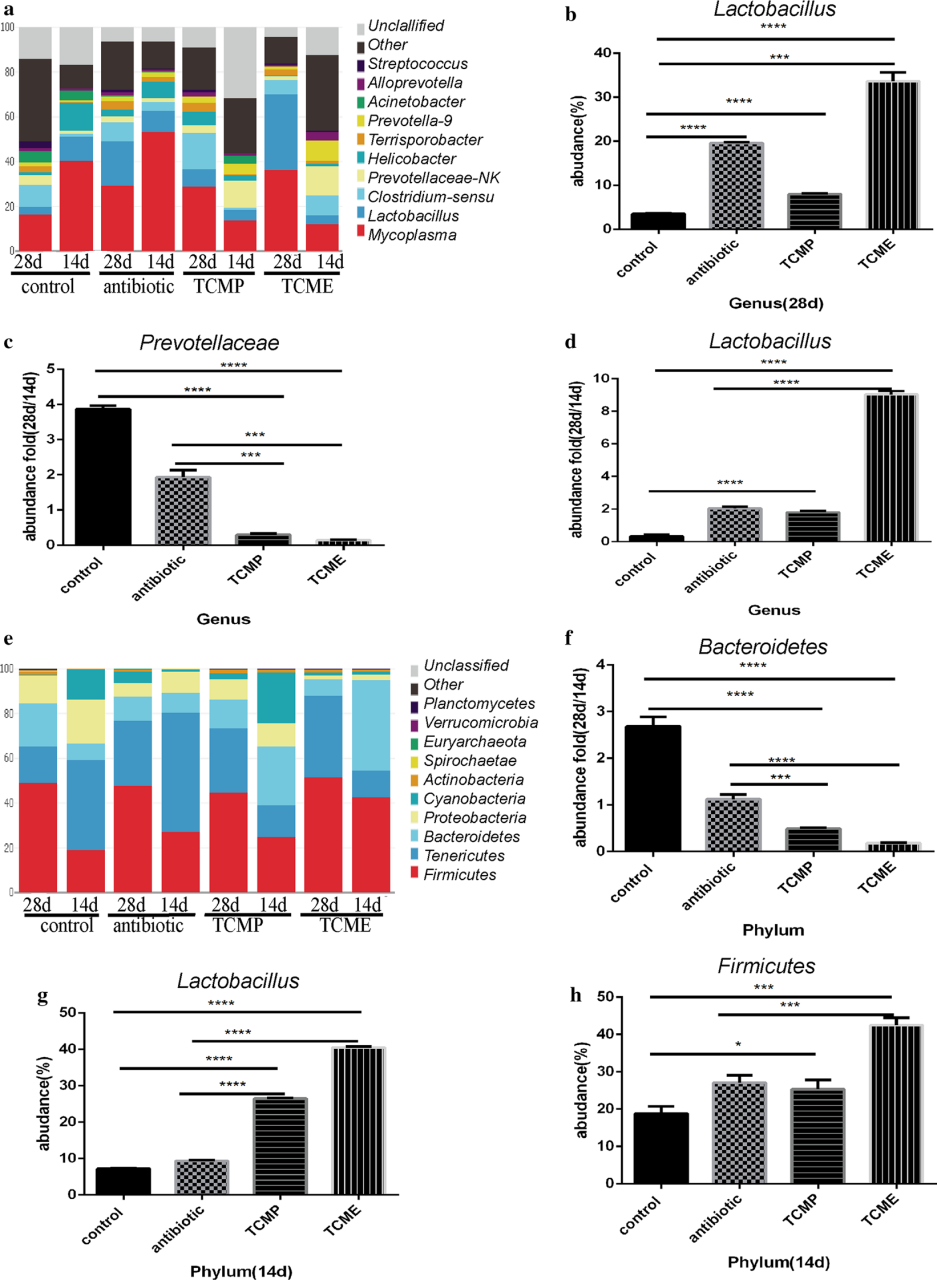
Effect of coix seed on the composition of jejunal microbiota with respect to genus and phylum. **a** Effect of coix seed on the composition of jejunal microbiota with respect to genus in weaned pigs. **b** Abundance of genus *Lactobacillus.* **c** Fold abundance of genus *Prevotellaceae* (28D/14D). **d** Fold abundance of genus *Lactobacillus* (28D/14D). **e** Effect of coix seed on the composition of jejunal microbiota with respect to phylum in weaned pigs. **f** Fold abundance of phylum *Bacteroidetes* (28D/14D). **g** Abundance of phylum *Lactobacillus*. **h** Fold abundance of phylum *Firmicutes* (14D). (*p < 0.05; **p < 0.02; ***p < 0.01, ****p < 0.001)

**Fig. 8:**
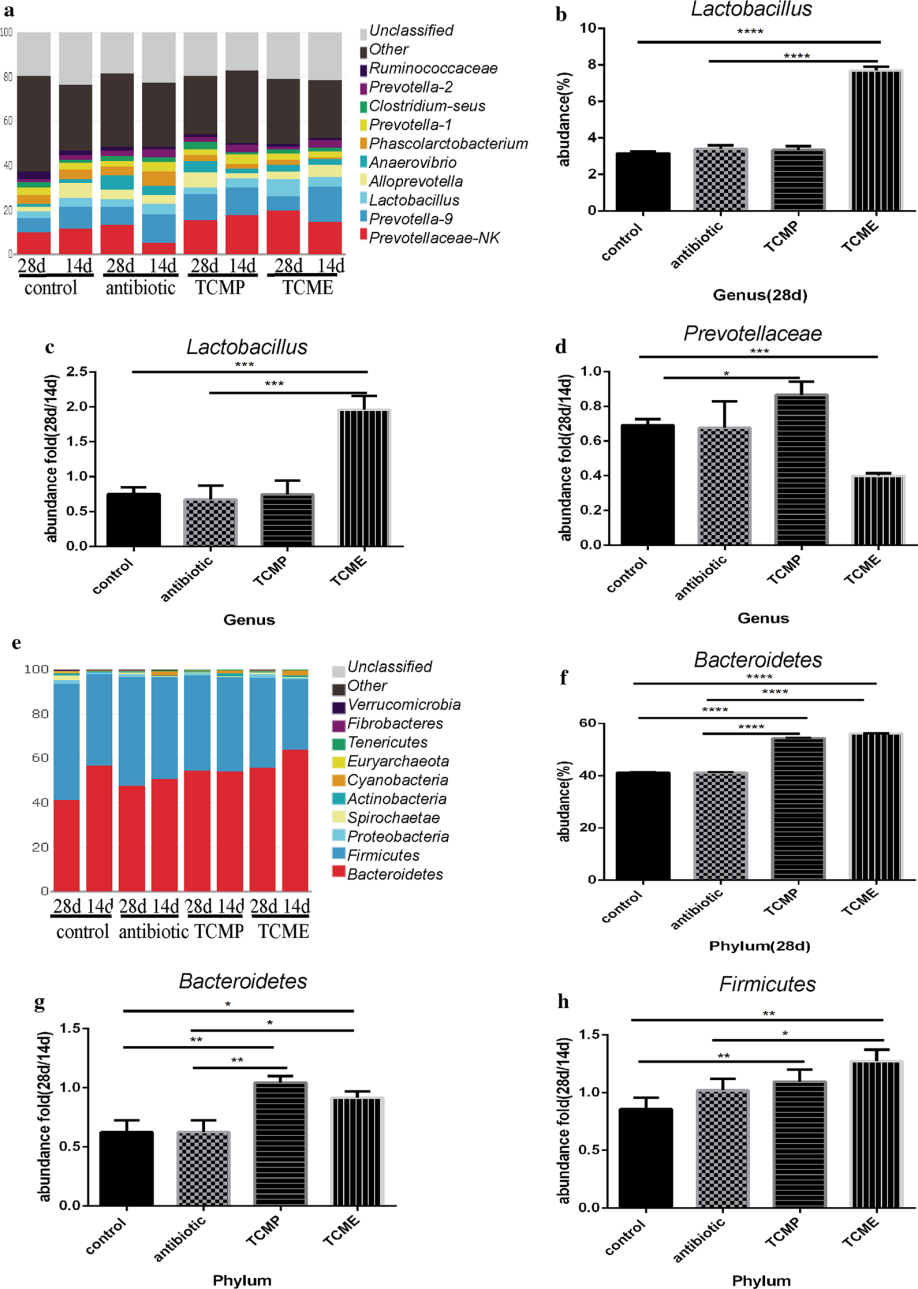
Effect of coix seed on the composition of colonic microbiota with respect to genus and phylum. **a** Effect of coix seed on the composition of colonic microbiota with respect to genus in weaned pigs. **b** Abundance of genus *Lactobacillus.* **c** Fold abundance of genus *Lactobacillus* (28D/14D). **d** Fold abundance of genus *Prevotellaceae* (28D/14D). **e** Effect of coix seed on the composition of colonic microbiota with respect to phylum in weaned pigs. **f** Fold abundance of phylum *Bacteroidetes* (28D/14D). **g** Abundance of phylum *Lactobacillus*. **h** Fold abundance of phylum *Firmicutes* (14D). (*p < 0.05; **p < 0.02; ***p < 0.01, ****p < 0.001)

In order to investigate the effect of coix seed on the composition of gut microbiota, we analyzed the bacterial populations of the succus gastricus, colon, ileum, and cecum on day 14 and day 28. The weaning pigs fed a coix seed diet showed a high relative abundance of the genus *Bactobaccillus* and phylum *Firmicutes* in the succus gastricus as compared to the other groups, with a significant increase from day 14 to day 28 (Fig. 5). Meanwhile, pigs supplemented with the extract of coix seed showed a clear decrease in abundance of genus *Prevotellaceae* and genus *Lactobacillus* in the ileum. The antibiotic group and control group showed no changes in genus *Prevotellaceae* in the ileum; simultaneously, the genus *Lactobacillus* in the ileum of both the antibiotic group and control group declined from day 14 to day 28 (Fig. 6). In weaning pigs fed with a coix seed diet, both genus and phylum *Bacteroides* in the colon showed higher abundance as compared to that in the antibiotic group and control group. From day 14 to 28, the genus *Bacteroides* in the colon showed an increase in TCMP and TCME, but was significantly decreased in the other groups (Fig. 7). We also observed that treatment with coix seed decreased the abundance of genus *Prevotella* and increased the abundance of genus *Bacteroides* in the cecum (Fig. 8).

## Discussion

Here, we demonstrated for the first time that feeding weaning pigs a coix seed diet can result in significantly improved growth and a reduced F:G. The coix seed diet also significantly reduced the pH of the succus gastricus, increased villus length, and significantly modulated the composition of gut microbiota.

Weaning pigs often are exposed to a multitude of stressors such as diet conversion, littermate separation, and disturbances in the surrounding environment, which frequently cause intestinal dysfunction, pathogen invasion, appetite decrease, feed intake decline, microecological imbalance, and growth retardation (Blecha and Kelley 1981; Funderburke and Seerley 1990; Ahrens et al. 2009; Hyun et al. 1998; Moeser et al. 2017). Therefore, maintaining normal gut health and microecological balance is vital to the growth performance of weaned pigs (Lallès et al. 2002). At present, antibiotics and additives have been widely used to alleviate the negative effects of weaning stressors (Lucas et al. 1959; Choi et al. 2011; Stahly et al. 1981). However, misuse and overuse of antibiotics increase antimicrobial resistance, which poses a serious threat of growing concern to human, animal, and environmental health (Hamscher et al. 2003; Sapkota et al. 2006; Murphy et al. 2007; Ferguson et al. 2016). Therefore, some herbal TCMs have been considered as substitutes for antibiotics. *Rhizoma Coptidis, Rhizoma Atractylodis*, *Pericarpium Citri Reticulatae*, *Fructus Mume, Fructus Psoraleae*, *Poria Cocos*, and *Semen Lablab Albumis* are commonly used for their anti-diarrheal or antimicrobial activity (Wang et al. 2017; Dong et al. 2007; Zhang 2005; Geng et al. 2011; Cheng et al. 2017; Fuchs et al. 2006). Coix seed is a plant material with considerable anti-insufficiency of spleen yang, anti-cancer, and anti-inflammatory properties (Han et al. 2017); it has also been shown to increase the immune response both in vitro and in vivo in human health (Liu et al. 2010; Kaneda et al. 1992; Tokuda et al. 1990; Qu et al. 2016; Yu et al. 2008). Moreover, use of coix seed in animal feed has shown some growth-promoting effects in cows (Luo et al. 2016), but the underlying mechanism has not been reported. Our results showed that coix seed actually improved growth performance and productivity post weaning. Furthermore, we found that coix seed significantly reduced the pH of the succus gastricus. Gastric acidity in piglets is known to promote the secretion and activity of digestive enzymes and plays a vital role in the process of piglet growth. Suckling piglets have relatively constant gastric acidity due to lactose fermentation, and thus dietary pH has little effect on their gastric pH. After weaning, the pigs lack a supply of lactose, which results in increased gastric pH, leading to gastrointestinal dysfunction and greater opportunity for pathogenic bacterial colonization. We found that a coix seed feed diet stabilized the gastric acidity in weaning pigs (pH 2–3.5), providing optimal activation conditions for pepsin in gastric juice (Bednarzewski 1968; de Gara et al. 1986). This increased activity of gastrointestinal digestive enzymes in weaned pigs further promotes the digestion and absorption of nutrients and improves growth performance and productivity. Additionally, lower gastrointestinal pH provides a favorable growth environment for *Lactobacillus* (Ramos et al. 2014). In our study, the coix seed group showed greater abundance of the genus and phylum *Lactobacillus* as compared with the antibiotic and control groups, whereas the abundance of genus *Prevotellaceae* in the ileum was low. Therefore, we conclude that coix seed might reduce gastric pH and promote pepsin secretion and the growth of *Lactobacillus* in piglets. However, the specific mechanisms require further research.

The integrity of gastrointestinal villus morphology is the premise of animal health, because it plays a critical role in nutrient digestion, absorption, and resistance to the invasion of pathogenic bacteria (Yang et al. 2013; Chen et al. 2008). However, at weaning, a carbohydrate-based diet damages the integrity of intestinal mucosa in piglets. In our control group and antibiotic group, the gastrointestinal villi showed emerging atrophy and disruption. This can largely be explained by the gastrointestinal epithelial cell damage and shedding caused by weaning. However, in the coix seed groups (TCMP and TCME), the gastrointestinal villi showed greater integrity and length. It is possible that the rich active components of esters, unsaturated fatty acids, sugars, and lactams in coix seed afforded sufficient nutrition for the growth of the intestinal villus epithelial cells.

It is well-established that gut microbiota affect host metabolism, induce metabolic disease, and play an important role in the health of animals and humans (Velagapudi et al. 2010; Cani et al. 2013). Gut microflora perform a wide variety of metabolic transformations, in which a variety of phytochemical compounds can be metabolized into products required for physiological activity (Eyssen 1973; Goldin 1990; Frick et al. 2012). Most oral medicines unavoidably affect gut microorganisms; however, traditional Chinese medicines are widely accepted as high-efficiency and low-toxicity “medicinal diets” that are capable of avoiding certain side effects and can be used to treat gastrointestinal discomforts by regulating microbiological balance (Chen et al. 2015). In this study, weaned pigs fed a coix seed diet showed a significant increase in *Lactobacillus* and *Bacteroides* in the gastrointestinal system, along with a decline in the abundance of *Prevotellaceae.* Because coix is similar to other cereals, starch is its main component, accounting for approximately 60%, and the content of oil, polysaccharides, and protein is higher than that in conventional cereals like rice and wheat; this makes coix a good culture medium for gut microbiota (Yang et al. 2011). In addition, the components of proteins and polysaccharides in coix seed play an important role in regulating water transport and afford a good growth environment for gut microbiota.

Previous studies have demonstrated that coix seed can increase the abundance of *Lactobacillus* and *Coprococcus*, induce imbalance of intestinal microbiota, and increase levels of probiotic bacteria (Caesar et al. 2015). Another report showed that coix seed enriches microbial metabolic pathways such as glycerolipid metabolism, biosynthesis of unsaturated fatty acids, the sulfate transport system, the manganese/iron transport system, and the glutathione transport system (Liu et al. 2019). Therefore, coix seed is a useful dietary supplement for the treatment of imbalanced gut microbial ecology (Wang et al. 2015). Thus, we speculated that coix seed would improve growth and reduce F:G in weaned pigs, which could be associated with increased amounts of *Lactobacillus* and *Bacteroides* and the enrichment of microbial metabolic pathways.

Collectively, the present study provides the first evidence that coix seed has beneficial effects on intestinal villus development and intestinal barrier function in weaned pigs, which might partly explain why the growth performance of pigs was improved by dietary coix seed supplementation.

## Data Availability

All data generated or analyzed during this study are included in this published article.

## References

[CR1] Ahrens F, Sünkel Y, Pollmüller T, Bussemas R, Weißmann F, Erhard M (2009). Influence of two different stressors, weaning and immunization, on the plasma histamine level of organic farming piglets. Inflamm Res.

[CR2] Bao K (2017). Non-scientific classification of Chinese herbal medicine as dietary supplement. Chin J Integr Med.

[CR3] Bednarzewski J (1968). Studies on the correlation between gastric acidity determined by titration and pH-metry and pepsin activity of the gastric juice, blood plasma and urine. Pol Tyg Lek.

[CR4] Blecha F, Kelley KW (1981). Effects of cold and weaning stressors on the antibody-mediated immune response of pigs. J Anim Sci.

[CR5] Caesar R, Tremaroli V, Kovatcheva-Datchary P, Cani P, Bäckhed F (2015). Crosstalk between gut microbiota and dietary lipids aggravates wat inflammation through TLR signaling. Cell Metab.

[CR6] Cani P, Everard A, Duparc T (2013). Gut microbiota, enteroendocrine functions and metabolism. Curr Opin Pharmacol.

[CR7] Centner T (2016). Recent government regulations in the United States seek to ensure the effectiveness of antibiotics by limiting their agricultural use. Environ Int.

[CR8] Chen X, Zhao J, Gregersen H (2008). The villi contribute to the mechanics in the guinea pig small intestine. Fed Am Soc Exp Biol J.

[CR9] Chen D-K, Li L-L, Zhang B, Zhang F-S, Li H-Q, Jiang J-J (2009). Effects of different additives on cherry volley duck growth performance and duck housemephitis concentration. Chinese J Ecol.

[CR10] Chen F, Wen Q, Jiang J, Li H-L, Tan Y, Li Y-H, Zeng N-K (2015). Could the gut microbiota reconcile the oral bioavailability conundrum of traditional herbs?. J Ethnopharmacol.

[CR11] Cheng X, Huo J, Wang D, Cai X, Sun X, Lu W (2017). Herbal medicine AC591 prevents oxaliplatin-induced peripheral neuropathy in animal model and cancer patients. Front Pharmacol.

[CR12] Choi JY, Shinde PL, Ingale SL, Kim J, Kim YW, Kim KH, Kwon IK, Chae BJ (2011). Evaluation of multi-microbe probiotics prepared by submerged liquid or solid substrate fermentation and antibiotics in weaning pigs. Livest Sci LIVEST SCI.

[CR13] Coffin R, Lin J, Scangas C et al (2013) Reducing antibiotic use in chinese pig farms. Gt Probl Semin Posters (All Posters, All Years), p 333. https://digitalcommons.wpi.edu/gps-posters/333

[CR14] de Gara C, Burget DW, Sivakumaran T, Hunt RH (1986). The effect of temperature and pH on the stability of human pepsin in stored gastric juice: a method to prevent activity loss. Scand J Gastroenterol.

[CR15] Dong H, Dong Y, He L (2007). Studies on constituents and anti-inflammatory activity of rhizoma Atractylodis macrocephalae. Chinese Pharm J.

[CR16] Eyssen H (1973). Role of the gut microflora in metabolism of lipids and sterols. Proc Nutr Soc.

[CR17] Ferguson D, Smith T, Hanson B, Wardyn S, Donham KJ (2016). Detection of airborne methicillin-resistant *Staphylococcus aureus* inside and downwind of a swine building, and in animal feed: potential occupational, animal health, and environmental implications. J Agromed.

[CR18] Frick J, Autenrieth IB (2012). The gut microflora and its variety of roles in health and disease. Curr Top Microbiol Immunol.

[CR19] Fu Y, Wang Y, Zhang B (2015). Systems pharmacology for traditional Chinese medicine with application to cardio-cerebrovascular diseases. J Tradit Chinese Med Sci.

[CR20] Fuchs SM, Heinemann C, Schliemann S, Härtl H, Fluhr J, Elsner P (2006). Assessment of anti-inflammatory activity of Poria cocos in sodium lauryl sulphate-induced irritant contact dermatitis. Skin Res Technol.

[CR21] Funderburke DW, Seerley RW (1990). The effects of postweaning stressors on pig weight change, blood, liver and digestive tract characteristics. J Anim Sci.

[CR22] Gao HX, Chen GS, Xu ZF (2010). Effects of compound Chinese herbal medicine on growth performance and blood biochemical index of weaning Xiangpig. Guizhou Agric Sci.

[CR23] Gellin G, Langlois BE, Dawson K, Aaron DK (1989). Antibiotic resistance of Gram-negative enteric bacteria from pigs in three herds with different histories of antibiotic exposure. Appl Environ Microbiol.

[CR24] Geng F, Wang W, Zhou T (2011). Antibacterial mechanisms of Fructus mume extract against *Listeria innocua*. Food Sci.

[CR25] Goldin B (1990). Intestinal microflora: metabolism of drugs and carcinogens. Ann Med.

[CR26] Hamscher G, Theresia Pawelzick H, Sczesny S, Nau H, Hartung J (2003). Antibiotics in dust originating from a pig-fattening farm: a new source of health hazard for farmers?. Environ Health Perspect.

[CR27] Han X, Ji X, Zhao H, Zhang Y, Liu G, Wang Y, Zhao W, Wang S (2017). on the mechanism of coix seed compositions in treatment of spleen deficiency and wet dampness Zheng. Afr J Tradit Complement Altern Med.

[CR28] He DY, Dai SM (2011). Anti-inflammatory and immunomodulatory effects of *Paeonia lactiflora* Pall., a traditional chinese herbal medicine. Front Pharmacol.

[CR29] Hsiao WLW, Liu L (2010). The Role of traditional chinese herbal medicines in cancer therapy—from tcm theory to mechanistic insights. Planta Med.

[CR30] Hui SY, Weng BC, Tu FL (2011). Effects of Chinese traditional herbal medicine complex supplementation on the growth performance, immunity and serum traits of pigs. Anim Sci J.

[CR31] Hyun Y, Ellis M, Riskowski G, Johnson RW (1998). Growth performance of pigs subjected to multiple concurrent environmental stressors. J Anim Sci.

[CR32] Jin LM, Qing-Lin WU, Jin BF, Fang GY, Liu HB, Lin ZP, Wang MH, Gao-Min MA, Dai H (2008). Influence of anti-heat stress Chinese herbal medicine additive on amount of milk and blood biochemical parameters in dairy cow. Chinese J Vet Sci.

[CR33] Jin R, Ye F, Yu QP (2011). Studies on the main nutrient components of Coat. Jilin Agric.

[CR34] Kaneda T, Hidaka Y, Kashiwai T, Tada H, Takano T, Nishiyama S, Amino N, Miyai K (1992). Effect of coix seed on the changes in peripheral lymphocyte subsets. Rinsho Byori.

[CR35] Lallès JP, Boudry G, Favier C, Le Floc’h N, Luron I, Montagne L, Oswald IP, Pié S, Piel C, Sève B (2002). Gut function and dysfunction in young pigs: physiology. Physiology.

[CR36] Lee HJ, Lee MH, Han IK (2000). Application of ELISA for the detection of penicillin antibiotic residues in live animal. Asian Aust J Anim Sci.

[CR37] Li L, Gong DQ, Zhang J, Chu DS, Liu ZH (2008). Effect of chinese herb medicine feed additive on immune performance of chinese yellow-feathered chicken. China Anim Husb Vet Med.

[CR38] Liu YQ, Liang J, Yang ZC, Liu BQ, Jin LM, Wz H (2010). Study progress on the pharmacological functions of Coix seed. J Anhui Agric Sci.

[CR39] Liu S, Li F, Zhang X (2019). Structural modulation of gut microbiota reveals Coix seed contributes to weight loss in mice. Appl Microbiol Biotechnol..

[CR40] Lucas IA, Calder AF, Smith H (1959). The early weaning of pigs IV. Comparisons of levels of antibiotic and sources of protein in diets for pigs weaned at 9 lb. live weight. J Agric Sci.

[CR41] Luo Z, Huang CG, Sha T (2016). Report on finishing experiment of coix seed shell on beef cattle. Rur Sci and Tech.

[CR42] Moeser A, Pohl C, Rajput M (2017). Weaning stress and gastrointestinal barrier development: Implications for lifelong gut health in pigs. Anim Nutr.

[CR43] Murphy M, Sanderson W, Vargo J (2007). Airborne antibiotic concentrations in a swine feeding operation. J Agric Saf Health.

[CR44] Normile D (2003). The new face of traditional chinese medicine. Science.

[CR45] Pettigrew J (2012) Health management with reduced use of antibiotics in pig production 9:130–138

[CR46] Qu D, Sun W, Liu M, Liu Y, Zhou J, Chen Y (2016). Bitargeted microemulsions based on coix seed ingredients for enhanced hepatic tumor delivery and synergistic therapy. Int J Pharm.

[CR47] Ramos C, Thorsen L, Ryssel M, Nielsen D, Siegumfeldt H, Schwan R, Jespersen L (2014). Effect of the gastrointestinal environment on pH homeostasis of *Lactobacillus plantarum* and *L. brevis* cells as measured by real-time fluorescence ratio-imaging microscopy. Res Microbiol.

[CR48] Sapkota A, Ojo K, Roberts M, Schwab KJ (2006). Antibiotic resistance genes in multidrug-resistant *Enterococcus* spp. and *Streptococcus* spp. recovered from the indoor air of a large-scale swine-feeding operation. Lett Appl Microbiol.

[CR49] Spiro DM, Welker MA, Arnold DH, Meckler GD (2011). A proposal to limit otoscopy to reduce unnecessary use of antibiotics: a call for research. Expert Rev Anti Infect Ther.

[CR50] Stahly TS, Cromwell GL, Monegue HJ (1981). Effects of the dietary inclusion of copper and(or) antibiotics on the performance of weanling pigs. J Anim Sci.

[CR51] Stein HH, Dong YK (2006). Reduced use of antibiotic growth promoters in diets fed to weanling pigs: dietary tools, part 2. Anim Biotechnol.

[CR52] Thexton AJ, Crompton A, German R (1998). Transition from suckling to drinking at weaning: a kinematic and electromyographic study in miniature pigs. J Exp Zool.

[CR53] Tokuda H, Matsumoto T, Konoshima T, Kozuka M, Nishino H, Iwashima A (1990). Inhibitory effects on Epstein-Barr virus activation and anti-tumor promoting activities of coix seed. Planta Med.

[CR54] Van Der Fels-Klerx HJ, Puister-Jansen LF, Van Asselt ED, Burgers SLGE (2011). Farm factors associated with the use of antibiotics in pig production. J Anim Sci.

[CR55] Velagapudi VR, Hezaveh R, Reigstad CS, Gopalacharyulu P, Yetukuri L, Islam S, Felin J, Perkins R, Borã©N J, Oresic M (2010). The gut microbiota modulates host energy and lipid metabolism in mice. J Lipid Res.

[CR56] Vente-Spreeuwenberg MAM, Beynen AC (2003) Diet-mediated modulation of small intestinal integrity in weaned piglets, Weaning the pig concepts & consequences, vol 7, pp 145–198

[CR57] Wang Q, Du Z, Zhang H, Zhao L, Sun J, Zheng X, Ren F (2015). Modulation of gut microbiota by polyphenols from adlay (*Coix lacryma-jobi* L. var. ma-yuen Stapf.) in rats fed a high-cholesterol diet. Int J Food Sci Nutr.

[CR58] Wang TT, Zhong LY, Ting XU, Pharmacy SO (2017). The influence on the antidiarrheal effect of mice and gastrointestinal motility by processing Coptidis Rhizome with fresh ginger juice, dry ginger juice and different ginger juice. Lishizhen Med Mater Medica Res.

[CR59] Weng CJ (2013). Effects of coix on growth performance of rabbits and pigs. Feed Expo.

[CR60] Williamson E, Lorenc A, Booker A, Robinson N (2013). The rise of Traditional Chinese Medicine and its materia medica: a comparison of frequency and safety of materials and species used in Europe and China. J Ethnopharmacol.

[CR61] Wolter BF, Ellis M, Corrigan BP, Dedecker J (2002). The effect of birth weight and feeding of supplemental milk replacer to piglets during lactation on preweaning and postweaning growth performance and carcass characteristics. J Anim Sci.

[CR62] Yang ZC, Liang J, Liu YQ (2011). Analysis of the constituents of Areaea chinensis. Anhui Agri Sci.

[CR63] Yang Huansheng, Xiong Xia, Yin Yulong (2013). Development and Renewal of Intestinal Villi in Pigs. Nutritional and Physiological Functions of Amino Acids in Pigs.

[CR64] Yu Y-L, Lu Y, Tang X, Cui F-D (2008). Formulation, preparation and evaluation of an intravenous emulsion containing brucea javanica oil and coix seed oil for anti-tumor application. Biol Pharm Bull.

[CR65] Zhang L-P (2005). Exploration on the benefit and function of *Pericarpium citri* reticulatae. Zhongguo Zhong Xi Yi Jie He Za Zhi.

[CR66] Zhang J, Zhang X, Liang X, Gu H, Zhu P (2008). Effects of different Chinese herbal medicines on biochemical parameters in guinea-pig with pigment gallstones. Zhong Xi Yi Jie He Xue Bao.

[CR67] Zhao L, Zhao A-G, Zhao G, Xu Y, Zhu X-H, Cao N-D, Zheng J, Yang J-K, Xu J-H (2014). Survival benefit of traditional chinese herbal medicine (a herbal formula for invigorating spleen) in gastric cancer patients with peritoneal metastasis. Evid Based Complement Altern Med.

[CR68] Zhou Z, Yang J, Kong A-N (2017). Phytochemicals in traditional chinese herbal medicine: cancer prevention and epigenetics mechanisms. Curr Pharmacol Reports.

